# Two-Dimensional Liquid Chromatography Method for the Determination of *Gelsemium* Alkaloids in Honey

**DOI:** 10.3390/foods11182891

**Published:** 2022-09-17

**Authors:** Xiao Ma, Meng-Ting Zuo, Xue-Jia Qi, Zi-Yuan Wang, Zhao-Ying Liu

**Affiliations:** 1 College of Veterinary Medicine, Hunan Agricultural University, Changsha 410128, China; 2 Hunan Engineering Technology Research Center of Veterinary Drugs, Hunan Agricultural University, Changsha 410128, China

**Keywords:** honey, two-dimensional liquid chromatography, koumine, humantenmine, *Gelsemium*, toxicity

## Abstract

Toxic Chinese medicine residues in honey pose a serious threat to consumer health. *Gelsemium* is one of the nine ancient poisons, making the whole plant virulent. The residue of *Gelsemium* alkaloid in honey causes poisoning from time to time. Therefore, it is very important to establish a method for the detection of *Gelsemium* alkaloids in honey. In this study, a method of solid phase extraction (SPE) with two-dimensional liquid chromatography (2D-LC) was developed for the first time for the simultaneous determination of *Gelsemium* alkaloids in honey, including gelsemine, koumine and humantenmine. First, the honey samples were purified by a PRS cation exchange column and extracted with 5% ammoniated methanol. Then, we verified the methodological indicators, which were in line with the Codex Guideline requirements. The verification results are as follows: matrix-matched calibrations indicated that the correlation coefficients were higher than 0.998. The recovery was in the range of 81%–94.2% with an intraday precision (RSD) of ≤5.0% and interday RSD of ≤3.8%. The limit of detection for the three alkaloids was 2 ng/g. The limits of quantification for gelsemine and koumine were 5 ng/g, and humantenmine was 20 ng/g. This method can be applied to the monitoring of *Gelsemium* alkaloids in honey.

## 1. Introduction

Honey is a natural sweet substance formed by mixing nectar or honeydew collected by bees and their own secretions [1]. It is a mixture of water and sugar and is rich in nutrients such as amino acids, minerals and essential trace elements and vitamins [2,3,4]. In our country, honey is a kind of medicine and food with a long history. “Shennong Materia Medica Classic” records that honey has many effects such as beneficial gas filling, pain relief and detoxification. Modern pharmacological studies have shown that honey has a variety of biological activities, such as anti-inflammatory, antioxidant, immune regulation and wound repair [5]. Due to its nutritional value and pharmacological activity, there is a great market demand. According to the data of the Food and Agriculture Organization of the United Nations, China is the world’s largest producer of bee culture and bee products, ranking first in the world in both the total number of bee colonies and the output of bee products [6]. There are about 300,000 beekeepers, 9.2 million bee colonies and about 500,000 tons of honey on the market [7]. However, some toxic substances are frequently introduced into honey products during the gathering honey process [8]. Therefore, the exact composition and contaminants in any batch of honey depend on the crops around the hives [9]. As early as 2000 years ago, many Greek soldiers accidentally ate the “mad honey”, resulting in a poisoning incident [10,11]. In recent years, in Yunnan, Guizhou and other places, incidents of accidentally eating toxic honey have also occurred [12,13,14]. It has been proven that honey poisoning is mainly caused by nectariferous plants, including *Gelsemium elegans, Common Threewingnut Root, Tripterygium hypoglaucum* and so on [15,16]. Therefore, it is very important to establish a method for the detection of toxic ingredients in honey.

*Gelsemium elegans* is a whole herb of the genus Mackeraceae, and it ranks first among the nine poisons in ancient China. Whole plants are highly toxic to nectariferous plants. According to their different distribution areas, the snout can be roughly divided into three categories. One is the *Gelsemium sempervirens,* mainly distributed in the south of the United States to Central America; another one is the *Asia Gelsemium*. Current research shows that a total of 121 indole alkaloids have been found in *Asia Gelsemium* [17]. According to the skeleton structure of the compounds, they are mainly divided into six categories of alkaloids, including gelsemine-type, koumine-type, gelsedine-type, humantenine-type, yohimbine-type and sarpagine-type alkaloids. According to a report, koumine has the highest content of indole alkaloids, which belongs to the koumine-type; this is followed by gelsemine, which belongs to the gelsemine-type. The most toxic indole alkaloid is humantenmine, which belongs to the most toxic alkaloid in *Gelsemium,* which belongs to the gelsedine-type [18,19,20]. In recent years, there have been many cases of poisoning caused by ingestion of *Gelsemium* honey. On the one hand, *Gelsemium* honey poisoning can lead to strong and rapid poisoning reactions, such as dyspnea and convulsions, and even death. On the other hand, there is no specific detoxification drug. Therefore, it is necessary to establish a rapid, sensitive method for the detection of the phytotoxin *Gelsemium* alkaloid in honey, Which it is very important for the prevention and diagnosis of *Gelsemium* poisoning.

At present, there are only methodological studies of gelsemine, koumine and humantenmine in biological and plant samples [21,22,23]. Detection methods for *Gelsemium* poisoning are also limited to biological samples, such as blood and urine [24,25]. However, there are few reports on alkaloid detection in honey. Recently, the solid-phase extraction method (SPE), QuEChERs method (Quick, Easy, Cheap, Effective, Rugged, Safe) and the liquid–liquid extraction (LLE) method have been used for the determination of toxic substance residues in honey. Honey is an extraordinarily complex matrix containing more than three hundred chemicals, and it is rich in proteins and lipids [26]. Each sample of honey has a specific set of twenty-five ingredients because it comes from a different plant [27]. Thus, honey substrates require complex and extensive pre-treatment to eliminate or reduce the matrix effects. The traditional LLE method has complicated steps and requires many expensive organic solvents. It takes 150–180 min to process a sample, and the extraction recovery rate of polar substances in honey is low [28]. Pau Calatayud et al. compared the effects of SPE, QuEChERs and LLE on the extraction recovery of 52 pesticides in honey, and the results showed that SPE was superior to QuEChERs in terms of precision and accuracy. In addition, the plasma effect of SPE was lower than that of QuEChERs [29]. Therefore, the SPE method has more advantages for the pretreatment of the honey matrix than other methods.

It has been reported that liquid chromatography–tandem mass spectrometry (LC–MS) was used to detect the alkaloids in honey, including gelsemine and koumine [30]. However, due to the cost of mass spectrometry and the high requirement for the laboratory, the general laboratory cannot meet the requirements. Recently, our team established a two-dimensional liquid chromatography (2D-LC) method for *Gelsemium* in biological samples [31]. On this basis, this study aims to establish a 2D-LC method for the simultaneous determination of three standards of *Gelsemium* alkaloids (gelsemine, koumine and humantenmine) in honey samples. This method provides a technical means for the detection of *Gelsemium* alkaloids in food species of animal origin and can be used for the rapid detection of nectariferous plant poisoning events and the traceability of alkaloids.

## 2. Materials and Methods

### 2.1. Chemicals and Reagents

The standard products, including gelsemine (CAS#: 1803-14-9, purity: 99.28%), koumine (CAS#: 1807-14-8, purity: 99.84%) and humantenmine (CAS#: 2106-30-8, purity: 99.63%), were purchased from Must (Chengdu, China). A PRS cation exchange column (500 mg, 3 mL) was purchased from Welch (Shanghai, China). A Waters Oasis HLB column (3 cc, 60 mg, 30 μm) was purchased from Waters (Shanghai, China). Methanol and acetonitrile of HPLC grade were purchased from Merck Co. (Darmstadt, Germany). Ammonium dihydrogen phosphate, phosphoric acid and ammonia were purchased from Kemiou (Tianjin, China). Ultra-pure water with a resistivity of <18.2 MΩ cm was prepared from a Milli-Q water purification system (Millipore, Bedford, MA, USA). The blank honey sample was purchased from various supermarkets. Thirty honey samples were collected from farmers’ markets in Hunan, Guizhou, Yunnan and Fujian Provinces from January 2022 to May 2022.

### 2.2. Preparation of Standard Solutions

Standard stock solutions of gelsemine, koumine and humantenmine were prepared with methanol at a concentration of 1 mg/mL and stored at −20 °C. The working solution of gelsemine, koumine and humantenmine was compounded by diluting the standard stock solution with methanol. Moreover, working solutions of gelsemine, koumine and humantenmine were added to blank honey to compound quality control (QC) samples. All QC samples were stored at −20 °C.

### 2.3. Sample Preparation Step

Honey (1.0 g) was mixed with 5 mL of water and vortexed for 2 min. Alkaloid was isolated using PRS cation exchange cartridges preconditioned with 5 mL of methanol and 5 mL of Milli-Q water. The mixed sample was passed through the PRS cartridges at a flow rate of less than 5 mL/min. Then, the PRS cartridges were rinsed with 10 mL of Milli-Q water and all effluent was discarded. The retained alkaloid was eluted with 8 mL of 5% ammoniated methanol. The eluate was evaporated to 0.5 mL using a gentle steam of nitrogen. Then, the sample was transferred quantitatively with methanol into a 1 mL volumetric flask, obtaining a final extract in 100% methanol. One milliliter of the extract was filtered through a 0.22-μm microbore cellulose membrane for 2D-LC analysis.

### 2.4. D-LC Analysis

The 2D-LC system was composed of an ACK 3200 column oven from ANAX with a fully automatic two-dimensional chromatographic coupling instrument (Changsha, China) and an LC-20AT high-performance liquid chromatograph from Shimadzu (Kyoto, Japan), which included a high-pressure LC-20AT pump (PUMP-B), two low-pressure gradient chromatography LC-20AT pumps (PUMP-A and PUMP-C), a CBM-20A system controller, a SIL-20A autosampler equipped with a 1000 μL injection loop and an SPD-20A UV detector.

The 2D-LC system used a heart-cutting transfer mode between the LC1 column and the LC2 column. The samples were first separated in 1D LC (LC1 column) mode to eliminate the interference of most impurities. The second step is the MC mode, in which the target material is transferred to the intermediate column after heart-cutting, where it is enriched. In the third step, the target was transferred to 2D LC (LC2 column) for further separation and, finally, UV detection. The mode switching in the whole process is done by LabSolutions LC Workstation V ER.5. The workstation corresponding to each mode can be highly automated to complete the corresponding work.

The first-dimensional (1D) liquid chromatography separation column was a strong cation exchange column (ASTON SXI 3.5 × 25 mm i.d.; particle size, 5 μm, Changsha, China). The middle column was a bonded phenyl column (SN-MA 4.6 × 10 mm i.d.; particle size, 5 μm, Changsha, China). The second-dimensional (2D) chromatography column was a reversed-phase column (SBR-2A 4.6 × 10 mm i.d.; particle size, 5 μm, Changsha, China). The mobile phase of PUMP-A was a 27:10:63 (*v*/*v*/*v*) solution of 80:20 (*v*/*v*) acetonitrile/water-10 mmol/L ammonium dihydrogen phosphate buffer (pH = 7.5)–10 mmol/L ammonium dihydrogen phosphate buffer (pH = 3.0). The PUMP-B mobile phase was water. The PUMP-C mobile phase was a 35:46:16.4:1.6:1 (*v*/*v*/*v*/*v*/*v*) solution of water–acetonitrile–methanol–85% phosphoric acid–acetic acid (pH = 7.0). The temperature of the column was maintained at 40 °C, and the injection volume was 500 μL. The flow rate of PUMP-A is 1.2 mL/min; the PUMP-C flow rate is 1 mL/min. The detector wavelengths were 254 nm and 263 nm.

### 2.5. Method Validation Procedure

The method was validated according to the Codex guidelines [32] and the parameters included linearity, specificity, accuracy, precision, limit of quantification (LOQ), limit of detection (LOD), stock solution stability and sample stability. For the calibration curve, linearity was evaluable by a seven-point detection matrix-matched calibration curve. For gelsemine and koumine, the concentrations were 5, 20, 50, 100, 200, 500 and 1000 ng/g; for humantenmine, the concentrations were 20, 40, 100, 200, 400, 800 and 1000 ng/g. The standard curve was constructed with an added concentration (X) as the abscissa and peak area (Y) as the ordinate.

Recovery was determined by four concentration added levels of QC samples, including QCLL (1 × LOQ), QCL (3 × LOQ), QCM (6 × LOQ) and QCH (30 × LOQ) (*n* = 6) detection. Precision is measured by intraday precision and interday precision, usually expressed as RSD values. Intraday and interday precision is the result of the repeated analysis of honey QC samples at four supplemental concentrations (*n* = 6) for one day (intraday precision) and three consecutive days (intraday precision).

The LOD and LOQ were calculated by preparing a sample of honey at the lowest concentration and calculating the S/N 3 and 10 times. The method specificity was evaluated by comparing multiple blank matrix samples with blank matrix spiked samples (blank honey matrix supplemented with gelsemine, koumine and humantenmine).

The standard stock solution stability was measured by the standard deviation (RSD) of four different time nodes, which were measuring the stock solution at −20 °C for 0 d, 7 d, 30 d and 60 d (*n* = 6). The working solution was diluted from the standard reserve solution to near the lower limit and upper limit of quantification, respectively.

The sample stability was measured by the standard deviation (RSD) of four honey samples at different concentration levels, which were freeze–thawed three times at room temperature (25 °C) for 24 h and frozen for 30 d at −20 °C (*n* = 6).

## 3. Results and Discussion

### 3.1. Optimization of Chromatographic Separation

The essence of the 2D-LC is through the LC1 flow of the target material and the matrix extraction column separation, which removes the impurities, then the elution target substances are enriched in the capture column for a further transfer, and then enter the LC2 flow path for detection through the analysis column. Among them, the interception window width of the LC1 flow path extraction column for the target substance and the LC2 peak separation degree are the key factors determining the transfer recovery rate, peak shape and time length of the two-dimensional analysis. This directly affects the accuracy, sensitivity and detection limit of the two-dimensional liquid chromatography detection method.

Our laboratory has previously established a two-dimensional liquid phase detection method for *Gelsemium* alkaloids in pig plasma, tissue and urine. Referring to the method described by Liu et al., we optimized the mobile phase and time program on this basis [31]. The interception window was 0.7–4.2 min when the mobile phase was an acetonitrile–phosphate solution. Excessive window width would cause a loss in the process of target material transfer, and more impurities would be transferred to the analysis column, resulting in a poor impurity removal effect. Eventually, the transfer recovery rate will be reduced, column efficiency will be reduced and the impurities will affect the target analysis. Only using methanol or acetonitrile and water as a mobile phase could not separate the three target substances well. Therefore, in order to adjust the viscosity and strength of the mobile phase and improve the separation effect and selectivity, we added methanol based on the original acetonitrile–water as the mobile phase for fine tuning, and the separation degree was improved, but the target peak tailing was relatively serious. Thus, to improve the peak shape, methanol acetonitrile water was used as the mobile phase, and 1.6% phosphoric acid (85%)–1% acetic acid was added to improve the peak shape and reduce tailing. However, the peak time of humantenmine is 0.6 min, which may lead to the failure of the interception of the target substance. Finally, the peak time of humantenmine was delayed by adding auxiliary water. Finally, the LC1 time program was determined to be 0–0.4 min with 1.2 L/min auxiliary water, and the interception window was 1.3–3.2 min. The LC2 mobile phase was adjusted to avoid the overlap of the peak times of the three target substances and to maintain a good separation degree. By adjusting the flow rate ratio of bottle-A (organic phase), bottle-B (water phase—alkali) and bottle-C (water phase—acid) in pump A, the peak time is delayed when the water phase increases, and the increase in the water phase—alkali ratio will lead to wider peak deformation. Therefore, by controlling the ratio of alkali and increasing the ratio of acid, the three target peaks can achieve a better separation and a good peak shape can be obtained. Finally, the flow rate ratio of pump-A was determined to be A:B:C = 27%:10%:63%. The optimized time program is shown in Table 1.

### 3.2. SPE Column Selection

**Figure 1:**
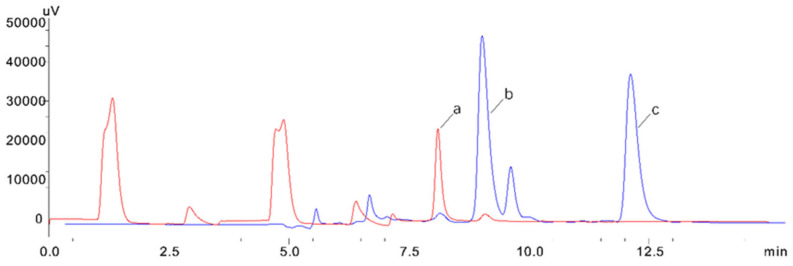
Comparison between the PRS cation exchange column (blue) and HLB solid phase extraction column (red). (**a**) Gelsemine, (**b**) koumine and (**c**) humantenmine.

We chose the solid phase extraction column (e.g., the HLB column and PRS cation exchange column) commonly used in alkaloid detection methods [33,34]. The HLB column is filled with a synthetic ultra-low pressure rapid reverse-phase chromatography filler, like the C18 column, which is suitable for the extraction of non-polar to moderately polar acidic, neutral and basic compounds. HLB columns are often used in the detection of complex substrates such as blood, urine and food. The PRS strong cation exchange column is based on the strong cation exchange being adsorbent. The main functional group is sulfonyl propyl, which has two ways of cation exchange and reverse phase retention. The retention mechanism is mainly related to strong cation exchange, while polarity is a secondary effect. It is suitable for the extraction of basic compounds. Therefore, we chose an HLB column and a PRS cation exchange column to investigate the effect of the pretreatment. Under the same pretreatment conditions (5 mL of methanol and 5 mL of Milli-Q water.), the HLB column showed poor retention of koumine and humantenmine, while the PRS column showed a high sensitivity and retention of koumine and humantenmine. The reason is that the cationic group of alkaloids can exchange with the positive ion [H] + of the PRS column, while the retention of the HLB column to the substance with greater polarity was poor. As shown in Figure 1, a cation exchange column was selected, and a solid phase extraction column (PRS) was used for sample extraction and purification; thus, a good peak shape and separation will be obtained.

### 3.3. Optimization Flush Solvent

**Figure 2:**
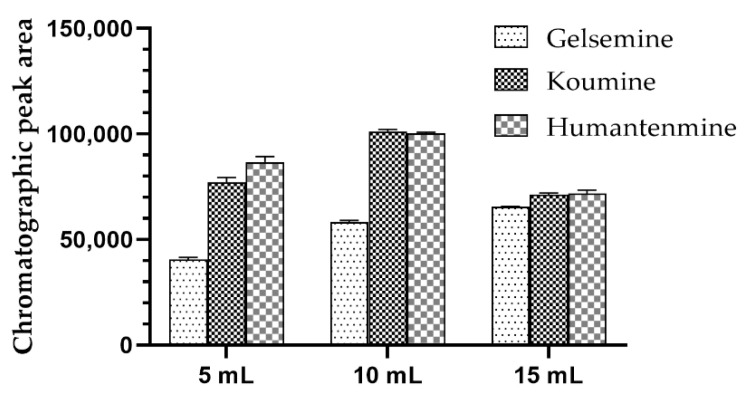
Comparison of the effects of 5 mL, 10 mL and 15 mL of flush solvent.

On the one hand, honey is a highly complex sugar mixture, and approximately 95% of the ingredients are sugar. On the other hand, the SPE column we used would adsorb substances with a larger polarity, so we chose to wash out the adsorbed impurities with water or methanol. Because the material is alkaloids, methanol leaching can lead to the loss of the target substance, reducing the extraction of the recovery. The sugar in honey is easily soluble in water. Therefore, water was selected as the flush solvent first, and then the amount of flush solvent was investigated. The results of rinsing with 5, 10 and 15 mL of water were investigated. The results are shown in Figure 2. The impurity removal effect of flushing with 15 mL of water is good, which is conducive to the recovery of gelsemine but will cause the loss of koumine and humantenmine, while flushing with 10 mL of water could completely elute saccharides and achieve a good recovery of the target components. However, the flushing effect of the 5 mL volume was significantly lower than that of the 10 mL volume. Combining the above results, 10 mL of water was selected to wash away the interference.

### 3.4. Optimization Elution Solvent

**Figure 3:**
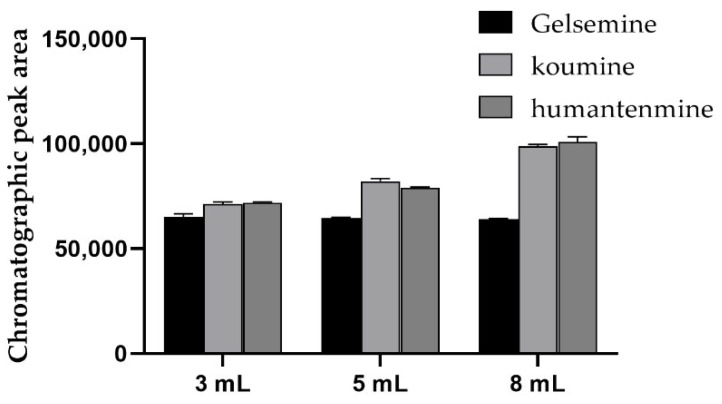
Comparison of elution effects with 3 mL, 5 mL and 8 mL of elution solvent.

Because the target substance is alkaloid, it was converted into a free alkaloid under alkaline conditions. The organic reagent can be made alkaline by adding the appropriate amount of ammonia water. We compared 1% ammoniated methanol with 5% ammoniated methanol. The eluting effect of the 1% ammoniated methanol was no different from that of the methanol. The eluting effect of the 5% aminoacylated methanol was obvious due to the 1% aminoacylated methanol. To better dissolve alkaloids in organic solvents, the 5% ammoniated methanol was selected for elution, and then the amount of elution solvent was investigated [30]. The target substance was eluted with 3 mL, 5 mL and 8 mL solvents. The effect of the elution solvent amount on the extraction recovery of the target substance was observed. With the increase in the amount of elution solvent, the recovery rate improved significantly. When the elution solvent was 3 mL, the recovery rate of leucosin and leucosin was about 65%, when the elution solvent was 5 mL, the recovery rate of leucosin and leucosin increased to 70%–75% and when the elution solvent volume reached 8 mL, the recovery rate was over 80%, which met the experimental requirements. The results are shown in Figure 3. More elution solvent can elute the target substance completely, but organic reagents are harmful to the body, so, after comprehensive consideration, 8 mL of the 5% ammoniated methanol was finally selected as the elution solvent in the spirit of cost savings and on the premise that the extraction recovery meets the experimental requirements.

### 3.5. Method Validation Results

**Figure 4:**
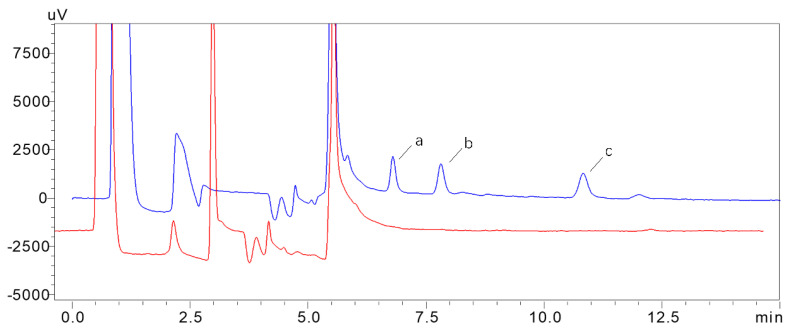
2D-LC of blank samples (red) and added sample (bule-20 ng/g) (**a**) gelsemine, (**b**) koumine and (**c**) humantenmine.

There was a good linear relationship between the concentration of gelsemine in honey and koumine in honey in the range of 5–1000 ng/g, and humantenmine in the range of 20–1000 ng/g. The linear correlation coefficients R2 were all higher than 0.998 when the injection amount was 500 μL. The extraction recoveries were in the range of 81%–94.2% with an intraday RSD, and the interday RSD were all less than 5%. The accuracy and precision parameters met the methodological requirements. The LOQ of gelsemine and koummine was 5 ng/g, and the LOQ of humantenmine was 20 ng/g (Figure 4). The LOD of gelsemine, koummine and humantenmine was 2 ng/g. In 2D-LC, the retention times of the three alkaloids were approximately 7.28 min, 8.30 min and 11.30 min. The peak shapes of the three alkaloids were good, and there was no interference near the target peak. The sample stability results were in the range of 1.1%–10.16% (Table 2). The standard stock solution stability results were in the range of 0.2%–2.3% (Table 3). The method has good linearity, recovery, accuracy, precision, durability and specificity. These results indicate that this method can be used for the detection of snout toxicity in honey samples, reduce the risk of snout poisoning and can also be applied to the clinical detection of honey poisoning events. What’s more, it also provides a theoretical basis and technical reference for the establishment of detection methods for other Chinese herbal medicines or toxic ingredients in honey.

### 3.6. Analysis of Actual Honey Samples

Thirty actual honey samples were collected from south China, where hookworm is widely distributed. Thirty honey samples were analyzed to assess the feasibility of the method. The results showed that there was no gelsemine, koumine or humantenmine residue in any of the 30 honey samples, which may be because our sampling time was from January to May, so we had not yet reached the full-bloom stage of the Gelsemium flower, or because the alkaloid content in the honey was very low and did not reach the detection limit of the method. In conclusion, according to our test results, there is a high probability that the honey on the market will not cause poisoning, but the overall evaluation of its safety needs more sample sizes and more test data of samples at different times.

### 3.7. Methods to Compare

Due to the high sensitivity and precision of LC-MS/MS, the current detection methods for toxic substances in honey mostly rely on LC-MS/MS but the laboratory requirements for LC-MS/MS are relatively high, so it is difficult to use and expensive to maintain. However, the long detection time and low sensitivity of liquid chromatography are not enough to meet the needs of detection. Two-dimensional liquid chromatography has the advantages of a short detection time, simple operating system, large injection volume and high sensitivity. Therefore, we compared the previously established LC-MS/MS and 2D-LC methods for simultaneous quantification of gelsemine, koumine and humantenmine in pig plasma and tissue. Some methods for detecting toxic alkaloids in honey include LC-MS/MS, HPLC and UHPLC-MS [30,35,36,37]. The 2D-LC method established by us for the simultaneous detection of gelsemine, koumine and humantenmine in honey has a slightly higher limit of quantification than LC-MS/MS, but it is superior to the two methods in accuracy, precision and stability.

## 4. Conclusions

In this study, a 2D-LC method for the simultaneous quantification of gelsemine, koumine and humantenmine in honey was established at first. It is optimized based on the SPE method, with a lower matrix influence, limit of quantitation and better accuracy and precision. This method has the advantages of a short detection time and high specificity. Compared with mass spectrometry and high-performance liquid chromatography, this method has a lower cost than mass spectrometry and a higher sensitivity than high-performance liquid chromatography. This method can be applied to the clinical detection and tracing of nectariferous plant poisoning. In summary, this method has wide application prospects in the clinical detection and tracing of nectariferous plant poisoning.

## Figures and Tables

**Table 1:** Time program for 2D-LC detection of three alkaloids.

*t*/min	Time Program Setting				
	0.00–1.20	1.20–3.20	3.21–3.8	3.81–4.7	4.70–17.0
Column connection	The 1D column is disconnected from the MC column	The 1D column is disconnected from the MC column	The 1D column is connected to the MC column	The MC column is connected to the 2D column	The MC column is disconnected from the 2D column
Major function	Complete sample on-line enrichment	Perform the first-dimension chromatography separation	The target component is transferred to the MC column	The target component is transferred to 2D column	The further two-dimensional separation of target components

**Table 2:** Results of linear range, correlation coefficient, LOD, recovery, accuracy, precision and sample stability.

Alkaloid	Added Concentration (ng/g)	Linear Range (ng/g)	Linearity (r2)	LOD (ng/g)	LOQ (ng/g)	Recovery (%)	Intraday RSD (%)	Interday RSD (%)	Short-Term RSD (%)	Freeze–Thaw RSD (%)	Long-Term RSD (%)
Gelsemine	QCLL	5–1000	0.9998	2	5	87.1	3.4	3.0	2.0	1.5	8.6
	QCL					94.2	1.9	2.0	1.2	4.5	3.1
	QCM					82.2	5.0	3.2	3.8	2.1	1.5
	QCH					83	3.6	3.8	2.4	1.8	4
Koumine	QCLL	5–1000	0.9985	2	5	92.9	3.4	2.0	2.9	1.2	4.3
	QCL					88.0	1.1	2.5	1.2	4.3	8.1
	QCM					89.4	3.8	3.8	5.4	5.8	10.2
	QCH					81	1.6	1.6	1.6	1.5	3.2
Humantenmine	QCLL	20–1000	0.9999	2	20	81.5	3.9	3.0	3.7	7.1	7.4
	QCL					82.8	3.8	3.5	1.1	2.1	5.5
	QCM					83.4	3.1	1.9	6.1	4.4	2.3
	QCH					81.3	2.3	2.6	1.5	2.4	2.4

**Table 3:** Results of standard stock solution stability.

Alkaloid	Added Concentration (ng/g)	0 d RSD (%)	7 d RSD (%)	30 d RSD (%)	60 d RSD (%)
Gelsemine	20	1.2	0.6	1.4	1.0
	900	0.4	1.0	1.5	1.3
Koumine	20	2.0	1.2	1.6	2.3
	900	0.9	0.6	1.1	1.7
Humantenmine	50	1.0	2.1	0.7	0.9
	900	0.2	1.2	1.6	2.0

## Data Availability

Data are contained within the article.
